# Cross-Protective IgG and IgA Antibodies against Oncogenic and Non-Oncogenic HPV Genotypes

**DOI:** 10.31557/APJCP.2020.21.9.2799

**Published:** 2020-09

**Authors:** Ana Paula Costa, Paulo César Giraldo, Ricardo Ney Cobucci, Márcia Lopes Consolaro, Raquel Pantarotto Souza, Luanda Barbara Canário, Paula Renata Machado, Rand Randall Martins, Pedro Vieira Baptista, José Eleutério Jr, Ana Katherine Gonçalves

**Affiliations:** 1 *Postgraduate Program in Health Sciences, Federal University of Rio Grande do Norte, Natal, Brazil. *; 2 *Department of Gynecology, and Obstetrics, State University of Campinas, Campinas, Brazil. *; 3 *Postgraduate Program in Biotechnology, Potiguar University, Natal, Brazil. *; 4 *Clinical Cytology and STD Laboratory, Department of Clinical Analysis and Biomedicine, State University of Maringá, Maringá, PR, Brazil. *; 5 *Department of Clinical Analysis and Toxicology, Federal University of Rio Grande do Norte, Natal, Brazil. *; 6 *Department of Pharmacy, Federal University of Rio Grande do Norte, Natal, Brazil.*; 7 *Hospital Lusíadas Porto and Unidade de Tracto Genital Inferior, Serviço de Ginecologia e Obstetrícia, Centro Hospitalar de São João, Porto, Portugal. *; 8 *Department of Female, Child and Adolescent Health, Federal University of Ceará, Fortaleza, Brazil. *

**Keywords:** Immunoglobulin G, immunoglobulin A, HPV, vaccine, cross protection

## Abstract

**Objective:**

The aim of the study was to describe the course of IgG/IgA immune response in women immunized with bivalent vaccine and in women non-vaccinated with HPV infection, as well as evaluating the cross-protection against non-vaccine HPV types.

**Methods:**

Serum and cervical mucus samples were collected from infected and vaccinated women for HPV detection/genotyping and for detection of IgG/IgA anti-HPV/VLP (Virus-like Particles) by ELISA.

**Results:**

The median absorbance detected in serum samples for anti-HPV-IgG antibodies was higher in vaccinated women when compared to HPV infected women (p <0.01), however, the median absorbance in cervical mucus samples for anti-HPV-IgA was higher in infected women when compared to vaccinated women (p<0.01). Additionally, our analyses also provided additional evidence for cross-protective efficacy of the HPV-16/18 vaccine against HPV-82, -6, -11, -13, -61, -72 and -74.

**Conclusion:**

The IgG antibodies were significantly more detected in the serum of vaccinated women, while the IgA was found in greater quantities in cervical samples from those infected by the virus. In addition, there is evidence that the bivalent vaccine provides cross-protection against other non-oncogenic viral subtypes.

## Introduction

Human papillomavirus (HPV) is the most common sexually transmitted infection of the female reproductive tract which can easily be spread through direct sexual contact and is associated with a variety of clinical conditions that range from innocuous lesions to cancer (Veiga et al., 2020). Different HPV types have been identified and classified as high-risk HPV (hrHPV) or low-risk HPV (lrHPV) based on their oncogenic potential (Woestenberg et al., 2018). HrHPV types 16 and 18 are associated with 71% of all cervical cancer cases, hrHPV types 31, 33, 35, 45, 52, and 58 with 21%, while lrHPV types, like 6 and 11 cause approximately 90% of anogenital warts (Artemchuk et al., 2019).

The first generation of vaccines targeted HPV16/18 (Cervarix^®^, GSK) or HPV6/11/16/18 (Gardasil®, Merk); more recently, a vaccine covering HPV 6/11/16/18/31/33/45/52/58 has been made available (Gardasil 9^®^, Merk) (Pinto et al., 2018). All available vaccines are based on non-infectious recombinant type specific L1 capsid proteins assembled into VLPs, acting as immunogens. These present an exterior surface closely mimicking HPV virions, and it is this multiplicity of L1 domains that stimulate a humoral immune response by exposing the system to VLPs, generating high neutralizing antibody titers 100 times higher than those occurring in natural infections (Gonçalves et al., 2016; Pinto et al., 2018).

HPV vaccines have demonstrated remarkable efficacy in phase III studies (Paavonen et al., 2007; Kreimer et al., 2011; Joura et al., 2015). They coincide with the induction of high affinity polyclonal anti-L1 IgG antibody response to the HPV types included in the vaccine, and 100% seroconversion in all targeted HPV types (Romanowski et al., 2016). 

The immunogenicity of vaccines differs from the immune response observed after natural infection. In this case, seroconversion is found in only a proportion of individuals following incident infection. Studies point out that many vaccines can elicit cross-reactive immune responses that may have an important additional clinical impact (i.e., cross-protection) on disease caused by non-vaccine strains or variants of the same pathogen. Cross-protection is likely to have beneficial effects on carriage and circulation, thereby providing some form of indirect protection (Pinto et al., 2018; Vojtek et al., 2019). 

The effectiveness of cross-protection against some non-vaccine types has been demonstrated for HPV-31/33/45 and, to a lesser extent, HPV 35, and for HPV 58 it was sustained and remained stable after 11 years post-vaccination (Wheeler et al., 2009; van der Weele et al., 2019; Tsang et al., 2020). Bivalent vaccine appears to confer greater cross-protection than other HPV vaccines and this difference is reflected in antibody levels against these non-vaccine types (Bissett et al., 2017). It has been shown that the bivalent induces the production of neutralizing immunoglobulin G/A (IgG/IgA) antibodies, which in turn play an important role in protecting against the HPV. Although HPV infects and propagates in the cervical mucosal epithelium and has almost no viremic phase, the ensuing humoral responses are most frequently detected in sera. However, immune responses in cervicovaginal secretions (CVS) are usually not investigated (Gonçalves et al., 2014; Pattyn et al., 2019). Knowledge about the cross-protection is important to understand the potential clinical impact of the bivalent HPV vaccination program.

Here, we provide direct information about the course of IgG/IgA antibody responses in CVS and serum of women with cervical intraepithelial neoplasia (CIN) and infected by HPV (presence of viral DNA) versus healthy women (immunized with bivalent vaccine (CEVARIX; GlaxoSmithKline Vaccines^®^).

## Materials and Methods

*Study Population*

We conducted a prospective study at a gynecological unit of a public university hospital. In this study, we enrolled 84 women, 35 vaccinated healthy women without HPV infection (DNA-HPV negative) and 49 HPV DNA-HPV positive/with HPV-induced intraepithelial lesion confirmed by cervical biopsy. Written informed consent was obtained from all participants. All procedures were carried out in compliance with the Declaration of Helsinki. All women were informed about the methods and objectives of the research and signed an informed consent form. The study was approved by the institutional Ethical Committee for Research (1.255.691/2015 CEP-HUOL).

Inclusion criteria were women vaccinated with 3 doses of the bivalent vaccine (Cervarix^®^, GSK) and women infected with HPV detected by PCR and not vaccinated. The exclusion criteria for both groups were: (1) any immunodeficiency, chronic illness, or treatment that could interfere with the immune response against HPV virus; (2) known systemic hypersensitivity to any components of the trial vaccine; (3) receipt of any other vaccine within four weeks; (4) menopausal status; (5) pregnant or breastfeeding; (6) use of hormonal contraceptives and (7) administration of immunoglobulins or blood products within three months before blood sampling. 

*Procedures*

Blood and cervical mucus sample were collected simultaneously. The aliquots of blood were collected into tubes containing separating gel and were coagulated at room temperature and centrifuged at 3,000 g to obtain the serum, then IgA/IgG levels were measured. The cervical samples were obtained by using a cytobrush, and after the collection from each participant the collection brush was placed in a tube containing PBS. The tube was kept at -20°C until processing for genomic DNA extraction.

*HPV detection and genotyping*

Genomic DNA was extracted and purified with the AxyPrep™ Body Fluid Viral DNA/RNA Miniprep Kit (Axygen, CA, USA®) according to the manufacturer’s instructions. HPV polymerase chain reaction (PCR) amplification was carried out using primers MY09 (5′-CGTCCMAARGGAWACTGATC-3′) and MY11 (5′-GCMCAGGGWCATAAYAATGG-3′) primers as described elsewhere (Manos et al., 1994). The PCR product was electrophoresed on a 1.5% agarose gel, stained with 1 μg/mL ethidium bromide, and photo-documented under UV light (approximately 450 bp). The samples that gave a positive PCR result were further analyzed by HPV genotyping. Two types of controls were also included in each reaction series: ‘no-DNA’ (negative control) and ‘HPV-positive DNA’ (positive control). 

HPV-positive samples were genotyped using a RFLP (Restriction Fragment Length Polymorphism) as described previously (Santiago et al., 2006). 10μg/mL of each PCR sample was digested with the restriction enzyme HpyCH4V (New England Biolabs, Ipswich, MA, USA) according to the manufacturer’s instructions. The genotyping was resolved on 8% polyacrylamide gels and was determined by analyzing each band with Labimage 1D software (Loccus Biotechnology, São Paulo, Brazil). Comparison of the molecular weights determined the genotypes following carcinogenic potential: HR-HPV, UR-HPV (undetermined-risk-HPV) and LR-HPV. 

*IgA and IgG anti-HPV-VLP detection by ELISA*

The initial antigen preparation has been described previously (Gonçalves et al., 2014). A plate of 96 wells was sensitized with 50μL of antigen (HPV-16/18 vaccine) diluted in carbonate-bicarbonate buffer (Sigma-Aldrich) at a concentration of 10μg/mL and incubated overnight at 4ºC. The plate was then washed with PBS-Tween 0.05% and blocked with 100μL of PBS with 10% of fetal bovine serum (FBS-Gibco) (PBS-FBS). The next step was incubation for 2h at room temperature and washing three times with PBS-Tween 0.05%. 

Cervical mucus and serum samples were diluted 1:100, 1:1,000, 1:10,000, 1:100,000, 1:1,000,000 and 1:10, 1:100, 1:1,000, 1:10,000, and 1:100,000, respectively in PBS-FBS, and 50μL of this dilution was added to each well. Following this, the samples were incubated for 2h at 37°C, and was washed with 0.05% PBS-Tween. Secondary antibody (peroxidase-labeled anti-human IgG or IgA; Sigma-Aldrich) was diluted 1:10,000 in FBS 10% and 50μL was added to the wells and incubated for 1h at 37ºC. After this, the plate was washed with PBS-Tween 0.05%, and then 50μL of substrate TMB (3,3´,5,5´-Tetramethylbenzidine Liquid Substrate System; Sigma-Aldrich) was added to the wells before incubating for 30 min at room temperature. The reaction was stopped with 50μL of 1N sulfuric acid, and the absorbance (optical density) of each well was read using an ELISA reader at 450nm with a reference filter of 630nm. The cutoff values were established from the ROC curve (Gonçalves et al., 2016), and for each antibody/sample the readings were as follows: IgG/serum, 0.616; IgG/mucus, 0.611; IgA/serum, 0.173; and IgA/mucus, 0.294.

*Statistical Analysis*

Statistical analysis was performed with Stata 11 (Stata Corporation, College Station, TX, USA). The different HPV genotype characteristics (high and low risk) in the women’s groups were described in relative and absolute frequency. To analyze the effect of vaccination on immunoglobulin concentration at different dilutions, linear regression of random effects was used. Serum and mucus immunoglobulin concentration was considered the dependent variable, dilution as a fixed effect the independent variable, and intra-individual variability as the random component of the model (p <0.05). In serum and mucus samples, mean absorbance values of IgG and IgA between HPV genotypes 16/18, 82, 6/11, and 13/61/72/74 were compared by analysis of variance followed by the Tukey’s post-test (p <0.05).

## Results

We enrolled a total of 84 patients divided into two groups, vaccinated (negative HPV-DNA) and unvaccinated (positive HPV-DNA) women. HPV infection was observed in 58.3% (49/84) of patients. 

**Figure 1:**
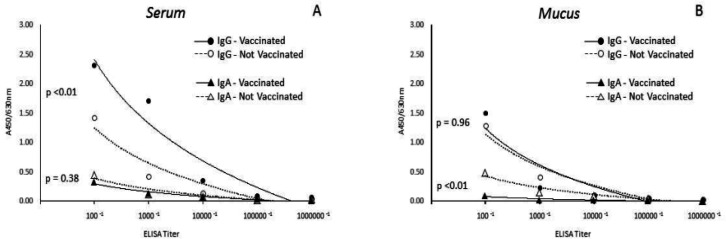
IgG/IgA Dilutions Regarding HPV-16/18 VLP in Serum and Mucus. A: The Median of Absorbance Detected in Serum Sample for IgG/IgA anti-HPV-VLP between vaccinated and not vaccineted women were P<0.01 and P= 0.38, respectively. B: The median of absorbance detected in mucus sample for IgG/IgA anti-HPV-VLP between vaccinated and not vaccineted women were P= 0.96 and P< 0.01, respectively

The ELISA assay for HPV-VLP (HPV-VLP-ELISA) was performed. It was observed that 100% of serum and mucus samples from both groups were positive for IgG/IgA antibodies when antigen was present. Positivity, however, decreases according to dilutions (Figure 1A/B).

The median absorbance detected in serum samples for anti-HPV-IgG antibodies was significantly higher in vaccinated women compared to unvaccinated women at 1:100, 1:1,000, 1:10,000, 1:100,000 and 1:1,000,000 dilutions, presenting statistical significance (P <0.01). In relation to anti-HPV-IgA antibodies in serum, there was no significant correlation between the groups (P = 0.38) (Figure 1A).

In the detection of anti-HPV-IgA, the median absorbance in cervical mucus samples was statistically higher in unvaccinated women compared to vaccinated women at 1:100, 1:1,000, 1:10,000, 1:100,000 and 1:1,000,000 dilutions (P<0.01), while the absorbance detected for anti-HPV-IgG under the same dilution conditions was not statistically significant (P = 0.96) (Figure 1B).

Of the 84 women studied for HPV DNA, 58.3% (49/84), the unvaccinated group, were positive for an HPV genotype and 41.6% (35/84). The vaccinated group were negative for HPV DNA. All HPV-positive samples were typed by RFLP. A total of 49 HPV DNA were detected, with 9 different genotypes. The overall frequency of HR and LR HPV types is shown in Table 1. 

**Figure 2:**
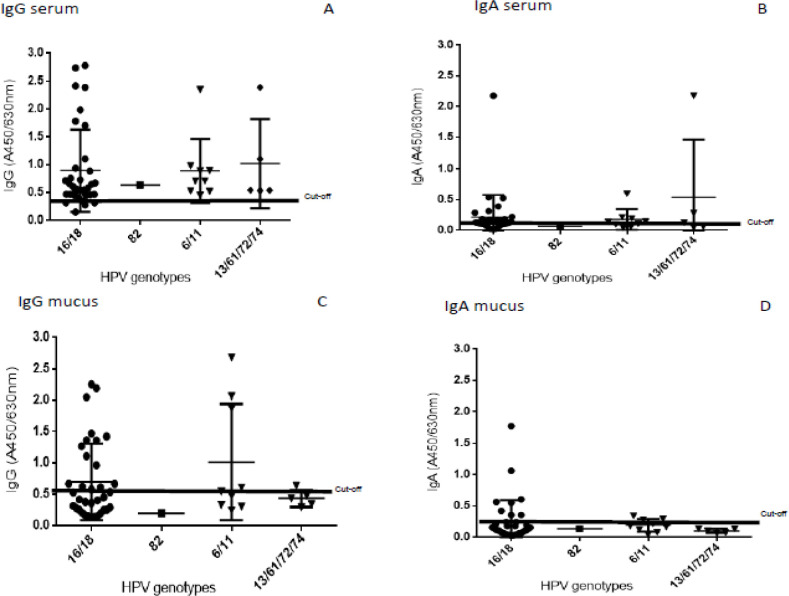
Absorbance of anti-HPV-IgG / IgA Antibodies in Serum and Cervical Mucus Induced by HPV Infection. A / B: IgG/IgA titers in serum samples. C/D: IgG / IgA Titers in cervical mucus samples

Naturally acquired anti-HPV IgG / IgA specific antibodies against a single HPV type were analyzed, although without statistical significance. A total of 41% (13/32) showed high concentrations of serum IgG antibodies for HPV16, 66% (2/3) for HPV18, 83% (5/6) for HPV6 and the other types such as HPV82/6/11/61/72/74 had positivity above 50% (Figure 2A). The percentages of mucus IgG detection were 44% (14/32) and above 50% for HPV 6 and 74 (Figure 2B). The IgA antibody positivity in serum was 31.2% (10/32) for HPV16 and 50% for HPV6 and 72 (Figure 2C). Regarding mucus IgA, positivity was 28% (9/32) for HPV16 and 33% for HPV6 (Figure 2D).

**Table 1:** The Frequency of HPV Genotypes, Including High-Risk (HR) and Low-Risk (LR) in HPV DNA-Positive Women

HPV genotype	Frequency	
	n	%
HR		
16	32	88.8
18	3	8.3
82	1	2.7
Total HR	36	100
LR		
6	7	53.8
11	1	7.6
13	1	7.6
61	1	7.6
72	2	15.3
74	1	7.6
Total LR	13	100
Total HPV DNA detected	49	100

## Discussion

In this study, cross-protection for HPV 6, 11, 13, 61, 72 and 74 (non-oncogenic virus) by the bivalent vaccine was present in the group of infected women. The IgG antibody was detected in greater quantities among vaccinated women, while IgA was significantly higher among those infected with non-immunized HPV. The high levels of IgG prove the efficacy of the bivalent vaccine by protecting against infection.

The presence of IgG antibodies is strongly associated with protection against cervical HPV infections (Pinto et al., 2018). As was seen in previous studies (Mollers et al., 2012; Scherer et al., 2016), the immune response detected after immunization indicated that the antibodies exhibited strong neutralizing activity (Mariz et al., 2020). Regarding the infected group, although (Pattyn et al., 2019) demonstrated that cervical HPV-specific antibodies from women with active HPV16 infections have some virus-neutralizing capabilities, the local HPV-specific IgA and IgG presence did not correlate with viral clearance and was not effective in inducing the regression of established lesions.

Concerning the IgA detection, this could be due to a possible transudation of the systemic circulation to the cervical mucosa, contributing to a protective environment in the cervix (Scherpenisse et al., 2013). However, low antibody levels in cervical mucus may be due to the shorter half-life of IgA when compared with IgG at this site, as well as the longer time required for antibody formation (Gonçalves et al., 2016), suggesting that antibody development following a natural infection can be a slow process and does not necessarily occur in all women (Carter et al., 2000). Probably, the IgA acts with mucosal immunity neutralizing intracellular viruses (Gonçalves et al., 2014) and preventing viral particles from infecting the cervical basal cell layer in the transformation zone (Pinto et al., 2018) and, as reported by Monroy et al., after natural infection, anti-HPV-IgA antibodies are detectable in the cervix, especially in persistent HPV infections (Monroy et al., 2010). 

Natural immunity to HPV is not as protective as vaccination (Beachler et al., 2016). Although the minimum antibody titer required for protection is not defined, recent studies (Safaeian et al., 2010; Castellsagué et al., 2014) have shown that anti-HPV antibodies acquired by natural infection provide protection if naturally acquired titers are high. Vaccination is not effective in patients with prevalent HPV infection, but the possible therapeutic benefit of the licensed HPV vaccines in reducing recurrent lesions in previously infected persons has been studied (Ghelardi et al., 2018; Pieralli et al., 2018; Stankiewicz Karita et al., 2019). The antibodies evoked by HPV vaccination during the follow-up post treatment for HPV-linked disease is recommended to reduce the risk of reactivation/re-infection or a new HPV infection, without adverse events or adverse reactions (Ghelardi et al., 2018; Pieralli et al., 2018).

The effector mechanism of the bivalent vaccine was mainly associated with a humoral response, as well as providing greater cross-protection over other HPV types (Kemp et al., 2011). The cross-protection of the HPV-16/18 vaccine was observed against persistent HPV-33, -31, -45, -51 and -52 associated infection in two studies, as well as highlighting a possible prophylactic effect against new infections and lesions (Wheeler et al. 2012; Jenkins, 2008). Although our study addresses other types of HPV (81, 6, 11, 13, 61, 72, and 74), the presence of antibodies can be explained by the fact that the effectiveness of cross-protection may differ across populations.

As far as we know, our study is the first to point out that the bivalent vaccine presents cross-protection against non-oncogenic viruses, in addition to comparing the immune response between unvaccinated and vaccinated women. However, some limitations should be addressed. First, our study presented a relatively small number of subjects, and the uneven distribution of CIN among these subjects may obscure the statistical significance. Secondly, there is a lack of a follow-up for some years to verify the maintenance of the immune response.

The IgG antibodies were significantly more detected in the serum of vaccinated women, while the IgA was found in greater quantities in cervical samples from those infected by the virus. In addition, there is evidence that the bivalent vaccine provides cross-protection against other non-oncogenic viral subtypes. Overall, we anticipate that there may exist substantial protection against cervical cancer and low-grade intraepithelial lesion over and above that achieved by HPV-16/18. Although further studies on the subject are needed, an expanded program on immunization is necessary to increase antibody titers and to reduce the risk of HPV infection and thus it can be argued that these immune mechanisms may provide protection against HPV.

## Author contribuitions

APC was responsible for the study conception, design, acquisition of data, analysis, interpretation of data and drafting of manuscript. PCG, JEJ, PVB and AKG were responsible for study conception, design, and critical revision. RNC was responsible for acquisition of data and drafting of manuscript. MLC, RPS, LBC and PRM were responsible for analysis and interpretation of data. RRM was responsible for analysis and interpretation of data and critical revision.

## References

[B1] Artemchuk H, Eriksson T, Poljak M (2019). Long-term antibody response to Human Papillomavirus Vaccines: Up to 12 years of follow-up in the Finnish Maternity Cohort. J Infect Dis.

[B2] Beachler DC, Jenkins G, Safaeian M (2016). Natural acquired immunity against subsequent genital Human Papillomavirus Infection: A Systematic Review and Meta-analysis. J Infect Dis.

[B3] Bissett SL, Godi A, Jit M (2017). Seropositivity to non-vaccine incorporated genotypes induced by the bivalent and quadrivalent HPV vaccines: A Systematic Review and Meta-Analysis. Vaccine.

[B4] Carter JJ, Koutsky LA, Hughes JP (2000). Comparison of human papillomavirus types 16, 18, and 6 capsid antibody responses following incident infection. J Infect Dis.

[B5] Castellsagué X, Naud P, Chow SN (2014). Risk of newly detected infections and cervical abnormalities in women seropositive for naturally acquired human papillomavirus type 16/18 antibodies: analysis of the control arm of PATRICIA. J Infect Dis.

[B6] Ghelardi A, Parazzini F, Martella F (2018). SPERANZA project: HPV vaccination after treatment for CIN2. Gynecol Oncol.

[B7] Gonçalves AK, Machado PR, Souza LC (2014). Detection of immunoglobulin IgA and IgG against human papilloma virus. Viral Immunol.

[B8] Gonçalves AK, Giraldo PC, Farias KJ (2016). Characterization of immunoglobulin A/G responses during 3 doses of the Human Papillomavirus-16/18 ASO4-adjuvanted vaccine. Sex Transm Dis.

[B9] Jenkins D (2008). A review of cross-protection against oncogenic HPV by an HPV-16/18 AS04-adjuvanted cervical cancer vaccine: importance of virological and clinical endpoints and implications for mass vaccination in cervical cancer prevention. Gynecol Oncol.

[B10] Joura EA, Giuliano AR, Iversen OE (2015). A 9-valent HPV vaccine against infection and intraepithelial neoplasia in women. N Engl J Med.

[B11] Kemp TJ, Hildesheim A, Safaeian M (2011). HPV16/18 L1 VLP vaccine induces cross-neutralizing antibodies that may mediate cross-protection. Vaccine.

[B12] Kreimer AR, González P, Katki HA (2011). Efficacy of a bivalent HPV 16/18 vaccine against anal HPV 16/18 infection among young women: A Nested Analysis Within the Costa Rica Vaccine Trial. Lancet Oncol.

[B13] Manos MM, Waldman J, Zhang TY (1994). Epidemiology and partial nucleotide sequence of four novel genital human papillomaviruses. J Infect Dis.

[B14] Mariz FC, Bender N, Anantharaman D (2020). Peak neutralizing and cross-neutralizing antibody levels to Human Papillomavirus types 6/16/18/31/33/45/52/58 induced by bivalent and quadrivalent HPV vaccines. NPJ Vaccines.

[B15] Mollers M, Scherpenisse M, van der Klis FR (2012). Prevalence of genital HPV infections and HPV serology in adolescent girls, prior to vaccination. Cancer Epidemiol.

[B16] Monroy OL, Aguilar C, Lizano M (2010). Prevalence of human papillomavirus genotypes, and mucosal IgA anti-viral responses in women with cervical ectopy. J Clin Virol.

[B17] Paavonen J, Jenkins D, Bosch FX (2007). Efficacy of a prophylactic adjuvanted bivalent L1 virus-like-particle vaccine against infection with Human Papillomavirus types 16 and 18 in young women: An Interim Analysis of a Phase III Double-Blind, Randomised Controlled Trial. Lancet.

[B18] Pattyn J, van Keer S, Tjalma W (2019). Infection and vaccine-induced HPV-specific antibodies in cervicovaginal secretions. A review of the literature. Papillomavirus Res.

[B19] Pieralli A, Bianchi C, Auzzi N (2018). Indication of prophylactic vaccines as a tool for secondary prevention in HPV-linked disease. Arch Gynecol Obstet.

[B20] Pinto LA, Dillner J, Beddows S (2018). Immunogenicity of HPV prophylactic vaccines: Serology assays and their use in HPV vaccine evaluation and development. Vaccine.

[B21] Romanowski B, Schwarz TF, Ferguson L (2016). Sustained Immunogenicity of the HPV-16/18 AS04-adjuvanted Vaccine Administered as a Two-Dose Schedule in Adolescent Girls: Five-year Clinical Data and Modeling Predictions From a Randomized Study. Hum Vaccin Immunother.

[B22] Safaeian M, Porras C, Schiffman M (2010). Epidemiological study of anti-HPV16/18 seropositivity and subsequent risk of HPV16 and -18 infections. J Natl Cancer Inst.

[B23] Santiago E, Camacho L, Junquera ML (2006). Full HPV typing by a single restriction enzyme. J Clin Virol.

[B24] Scherer EM, Smith RA, Gallego DF (2016). A single Human Papillomavirus vaccine dose improves B cell memory in previously infected subjects. EBioMedicine.

[B25] Scherpenisse M, Mollers M, Schepp RM (2013). Detection of systemic and mucosal HPV specific IgG and IgA antibodies in adolescent girls one and two years after HPV vaccination. Hum Vaccin Immunother.

[B26] Stankiewicz Karita HC, Hauge K, Magaret A (2019). Effect of Human Papillomavirus Vaccine to Interrupt Recurrence of Vulvar and Anal Neoplasia (VIVA): A Trial Protocol. JAMA Netw Open.

[B27] Tsang SH, Sampson JN, Schussler J (2020). Durability of cross-protection by different schedules of the bivalent HPV Vaccine: The CVT Trial. J Natl Cancer Inst.

[B28] van der Weele P, Breeuwsma M, Donken M (2019). Effect of the bivalent HPV vaccine on viral load of vaccine and non-vaccine HPV types in incident clearing and persistent infections in young Dutch females. PLoS One.

[B29] Veiga CRP, Semprebon E, Silva JL (2020). Facebook HPV Vaccine Campaign: Insights From Brazil. Hum Vaccin Immunother.

[B30] Vojtek I, Buchy P, Doherty TM (2019). Would immunization be the same without cross-reactivity?. Vaccine.

[B31] Wheeler CM, Castellsagué X, Garland SM (2012). Cross-protective efficacy of HPV-16/18 AS04-adjuvanted vaccine against cervical infection and precancer caused by non-vaccine oncogenic HPV types: 4-year end-of-study analysis of the randomised, double-blind PATRICIA trial. Lancet Oncol.

[B32] Wheeler CM, Kjaer SK, Sigurdsson K (2009). The impact of quadrivalent Human Papillomavirus (HPV; Types 6, 11, 16, and 18) L1 virus-like particle vaccine on infection and disease due to oncogenic nonvaccine HPV types in sexually active women aged 16-26 years. J Infect Dis.

[B33] Woestenberg PJ, King AJ, van Benthem BHB (2018). Bivalent vaccine effectiveness against type-specific HPV positivity: Evidence for Cross-Protection Against. J Infect Dis.

